# Optimizing Epoch Length and Activity Count Threshold Parameters in Accelerometry: Enhancing Upper Extremity Use Quantification in Cerebral Palsy

**DOI:** 10.3390/s24041100

**Published:** 2024-02-08

**Authors:** Isabelle Poitras, Léandre Gagné-Pelletier, Jade Clouâtre, Véronique H. Flamand, Alexandre Campeau-Lecours, Catherine Mercier

**Affiliations:** 1 Centre for Interdisciplinary Research in Rehabilitation and Social Integration (Cirris), Centre Intégré Universitaire de Santé et Services Sociaux de la Capitale-Nationale, Quebec City, QC G1M 2S8, Canada; isabelle.poitras.2@ulaval.ca (I.P.); leandre.gagne-pelletier.1@ulaval.ca (L.G.-P.); jade.clouatre.1@ulaval.ca (J.C.); veronique.flamand@fmed.ulaval.ca (V.H.F.); alexandre.campeau-lecours@gmc.ulaval.ca (A.C.-L.); 2 School of Rehabilitation Sciences, Laval University, Quebec City, QC G1V 0A6, Canada; 3 Department of Mechanical Engineering, Laval University, Quebec City, QC G1V 0A6, Canada

**Keywords:** cerebral palsy, accelerometer, upper extremity, movement quantification, thresholds, epoch length, bimanual, activity monitoring

## Abstract

Various accelerometry protocols have been used to quantify upper extremity (UE) activity, encompassing diverse epoch lengths and thresholding methods. However, there is no consensus on the most effective approach. The aim of this study was to delineate the optimal parameters for analyzing accelerometry data to quantify UE use in individuals with unilateral cerebral palsy (CP). Methods: A group of adults with CP (*n* = 15) participated in six activities of daily living, while a group of children with CP (*n* = 14) underwent the Assisting Hand Assessment. Both groups performed the activities while wearing ActiGraph GT9X-BT devices on each wrist, with concurrent video recording. *Use ratio* (*UR*) derived from accelerometry and video analysis and accelerometer data were compared for different epoch lengths (1, 1.5, and 2 s) and *activity count* (*AC*) *thresholds* (between 2 and 150). Results: In adults, results are comparable across epoch lengths, with the best *AC thresholds* being ≥ 100. In children, results are similar across epoch lengths of 1 and 1.5 (optimal *AC threshold* = 50), while the optimal threshold is higher with an epoch length of 2 (*AC* = 75). Conclusions: The combination of epoch length and *AC thresholds* should be chosen carefully as both influence the validity of the quantification of UE use.

## 1. Introduction

Quantifying upper extremity (UE) use in neurologically impaired populations has become increasingly popular in rehabilitation research [1,2,3,4,5]. One way to quantify UE use in daily life is to use self-report questionnaires such as the Motor Activity Log and the Rating of Everyday Arm-Use [6,7]. One of the limitations of these assessments is their subjective nature, which introduces biases such as recall bias and desire for social approval [8]. These biases may be exacerbated by the characteristics of populations with neurological deficits such as cognitive, memory, and language deficits [9,10]. Alternatively, wrist-worn accelerometers allow the objective quantification of UE use in daily life. They have been validated in healthy subjects and in several populations with neurological disorders (i.e., concurrent validity and inter-rater reliability), including stroke and cerebral palsy (CP) [11,12,13,14]. Most commercially available movement quantification devices measure movements (accelerations) in three axes and convert them into arbitrary units called *activity counts* (*AC*) over a predefined time epoch. Over the years, diverse protocols have been used to quantify the use of the UE with accelerometers, involving varying sampling frequencies, epoch lengths, and thresholding methods. This lack of standardization in methods has been criticized by several authors [14,15] and limits the possibility of comparing results across studies. A consensus on the most effective approach to data processing has yet to be reached.

Among the methods that are most widely used is the one developed by Uswatte and colleagues, where a threshold of ≥2 *AC* per 2 s epochs is applied to determine the presence of movement during this period [16]. One of the problems with this method is that the algorithm used to calculate *AC* varies across studies, depending on the accelerometers used (i.e., the algorithms of most commercially available devices are not shared with users). This variation can have a significant impact on *AC*, and consequently, on the threshold used for *AC*. However, this approach has been used in the literature with some variations from one author to another, such as thresholds of ≥2 *AC* [17,18], ≥1 *AC* [19,20], or >0 *AC* [21] on 1 s epochs, or a ≥1 *AC* on a 2 s epoch [22,23], without justification for these arbitrary changes. The current threshold of 2 *AC* has recently been questioned on the grounds that such a low threshold would only discriminate periods of absolute stillness, thus including a large proportion of small non-functional movements, leading to a significant overestimation of functional UE use. A recent study aiming to identify an optimal threshold in stroke subjects showed that a higher threshold (≥20.1 *AC* for the more affected (MA) side and ≥38.6 *AC* for the less affected (LA) side) had a better accuracy than the traditional threshold of 2 *AC* [24]. While very interesting from a methodological perspective, setting an arm-specific threshold in each patient is currently not applicable to clinical practice. An experiment conducted by our team also demonstrated the validity of a higher threshold of ≥100 AC per 1 s epoch in adults with unilateral CP [13] when aiming to characterize various unimanual and bimanual metrics, including the *use ratio* (*UR*) which has been widely used in clinical studies [14].

Likewise, wide variations in epoch length are observed in the literature, with epochs of 1 s [17,18,19,20,21], 2 s [16,23], 15 s [25,26], 60 s [27,28], or 120 s [29], often without any justification for the choice of epoch. Experts in the field have recommended the use of 1 s epochs [15,30]. However, the impact of epoch length on quantification of UE use has never been formally demonstrated, and some evidence based on the physical activity literature shows that epoch length has a significant impact on movement quantification, with longer epochs tending to overestimate movement in adults [31] but to underestimate physical activity in children [32].

The aim of this study was to delineate the optimal parameters for analyzing accelerometry data to quantify UE use in individuals with unilateral CP, spanning both adults and children. Specifically, we characterized the effects of epoch length and *AC threshold* on the measure of *UR*. The *UR* derived from accelerometry data was compared to the one derived from video analysis.

## 2. Materials and Methods

### 2.1. Participants

Two groups of participants were recruited for this study: a group of adults with CP (Adult Group) and a group of children with CP (Children Group). The Adult Group had previously been studied in [13]. The Adult Group was recruited through the Université Laval mailing list and by consulting health records of the Centre intégré universitaire de santé et de services sociaux de la Capitale-Nationale (CIUSSS-CN). The inclusion criteria for the Adult Group were as follows: (1) being 18 years of age or older; (2) having a diagnosis of unilateral spastic CP; and (3) having mild to moderate UE impairments (i.e., being categorized as level I, II, or III on the Manual Ability Classification Scale [MACS]). The exclusion criteria were as follows: (1) having significant visual or cognitive impairments that will interfere with doing the task; and (2) needing help to walk or to control a wheelchair. The Children Group was recruited through the Université Laval mailing list and through the CIUSSS-CN. The inclusion criteria were as follows: (1) being aged between 7 and 18 years of age; (2) having a diagnosis of unilateral spastic CP; and (3) having mild to moderate UE impairments (i.e., being categorized as MACS I, II, or III). The exclusion criterion was as follows: (1) having significant visual or cognitive impairments that will interfere with participating in the camp. The two projects were approved by the local ethics committee (i.e., Ethics #2018-609, CIUSSS-CN for the Adult Group, and Ethics #2020-1961, CIUSSS-CN for the Children Group). For the Adult Group, each participant provided written consent, while for the Children Group, the written consent of the child’s legal guardian was obtained. The motor deficits exhibited by participants were described using a clinical assessment, the Jebsen Taylor Hand Function, which evaluates unimanual motor function. It consists of seven tasks (1—writing; 2—turning cards; 3—picking up small objects; 4—simulating feeding, 5—stacking tokens; 6—lifting light objects; and 7—lifting heavy objects. The writing subtest was not performed with children [33]. The performance was measured in seconds, and then converted into a *z-score* based on the normative value by age [33,34], allowing for the identification of UE impairments (impaired if *z-score* > 1.96).

### 2.2. Experimental Setup

**Figure 1:**
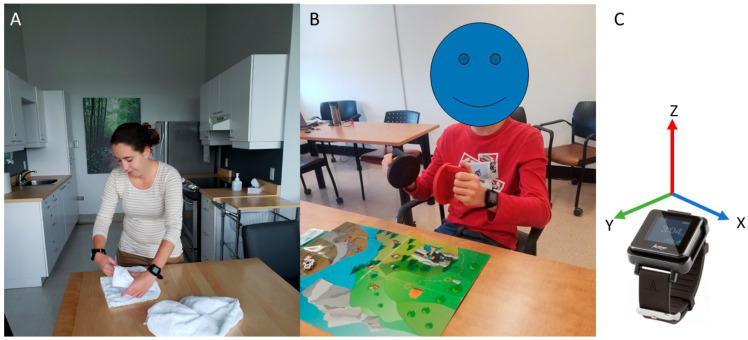
An example of the experimental setup for each task. Panel (**A**) shows one of the bilateral tasks (i.e., folding towels) performed by participants of the Adult Group, and Panel (**B**) shows one of the tasks performed by children during the Assisting Hand Assessment. Both participants wore one ActiGraph GT9X-BT (illustrated on Panel (**C**)) on each wrist during the tasks.

Both groups performed experimental tasks involving bilateral UE activities while wearing an ActiGraph GT9X-BT (see Figure 1 for experimental setup, sample rate = 100 Hz, internal memory of 4Go, accelerometer dynamic range of ±8 g, Actigraph LLC, Pensacola, FL, USA) on each wrist, with the sessions being recorded on video (recorded at 30 frames/second). The participants performed three shoulder flexion movements at the beginning of each task or play session to help offline data processing (i.e., synchronizing accelerometry data and video recording). The experimental tasks differed between the two groups.

Adults with CP performed six tasks (1—cleaning the table; 2—making a coffee; 3—setting up the table; 4—pouring a glass of water; 5—folding towels; 6—putting toothpaste on a toothbrush). The tasks were chosen to cover a variety of bilateral and unilateral UE uses. The tasks and the discriminative value of the tasks were already described in [13].

Children with CP performed the Assisting Hand Assessment. The Assisting Hand Assessment consists of a short play session (~10 to 20 min) that allows for assessing the integration of the MA hand in bimanual activities, while seated at a table [35].

### 2.3. Video Processing

The video data underwent processing using specialized Python software (Python Software Foundation, Wilmington, DE, USA; Python Language Reference, version 3.7), allowing for a 25% reduction in the original video playback speed. The evaluator was instructed to identify ‘arm movements’, defined as visually detectable wrist displacements. Within the Python software, the slow-motion video was presented to the evaluator, who pressed the space bar when the arm was in motion, assigning a value of one to the timeframe. When the button was released, a value of 0 was allocated. This process was performed for each arm separately. The ratings obtained from video processing were then interpolated to generate data equivalent to 100 frames/second, enabling a comparison with accelerometry data. In the case of the Adult Group, the inter-rater reliability of the quantification method had already been demonstrated in [13] leading to only one evaluator (IP) processing the video. However, inter-rater reliability was not previously assessed in children performing the Assisting Hand Assessment. Therefore, two of the authors (I.P. and L.G.P.), who are both occupational therapists with >4 years of experience, processed the video independently for the Children Group. Inter-rater reliability was found to be high (*ICC* = 0.93, *p* < 0.001), indicating excellent agreement between the two evaluators. Therefore, ratings from I.P., who rated videos from both the Adult and Children Groups, were used for subsequent analyses.

### 2.4. Accelerometry Processing

The pre-processing of accelerometry data was carried out using ActiLife 6 software (Actigraph LLC, Pensacola, FL, USA, sampling rate of 100 Hz) to obtain raw data. A custom Matlab program (MATLAB version 9.6.0 (R2022b), Natick, MA, USA, The MathWorks Inc.) was used to perform the offline data processing by filtering through a continuous eighth-order bandpass filter and transforming raw data into *AC*; see details in [36]. Then, the same custom Matlab program was used to compute activity counts and accelerometry metrics and to process data through epochs of, 1 s, 1.5 s, and 2 s. The activity counts calculation was based on the vector magnitude (*AC*$=\sqrt{\;{s_{xi}}^{2}+{s_{yi}}^{2}+{s_{zi}}^{2}}$) representing the norm of the activity counts on the three axes *x*, *y*, and *z* ($s_{xi}$, $s_{yi}$, and $s_{zi}$ denote the sum of *AC* for a specific epoch). Firstly, the *AC* was calculated for each arm separately. Afterward, the software identified the presence of arm movements by allocating a value of 1 (i.e., movement) when reaching a specific *AC*, and a value of 0 (i.e., no movement) when failing to exceed the *AC threshold*. To compare the effect of *AC threshold* levels, we used three different *AC thresholds*: the commonly used *AC threshold* of 2 [16], the previously validated *AC threshold* for adults with CP (100) [13], as well as five additional *AC threshold* values between the two (25, 50, 75) and above (125, 150).

### 2.5. Use Ratio Calculation

To measure the use of both hands during the two different tasks, the *UR* was calculated. The *UR* represents the proportion of the epoch where the MA arm is used versus the LA arm (*UR* > 1 represents more MA arm use; *UR* < 1 represents more LA arm use; and UR = 1 represents equal use). This can be calculated as follows:

(1) $$UR=\frac{movement\;duration\;LA\;arm\;({AC}_{LA}\;\neq 0)}{movement\;duration\;MA\;arm\;({AC}_{MA}\;\neq 0)}$$

where ${AC}_{MA}\neq 0$ represents an epoch where the MA arm is moving and ${AC}_{LA}\neq 0$ represents an epoch where the LA arm is moving.

### 2.6. Statistical Analysis

The difference between the *UR* derived from accelerometry data and the one derived from video analysis (Δ*UR*) was calculated for each participant, each epoch length, and each AC threshold. Mean and 95% confidence interval (95%CI) were computed for each combination of parameters. A 95%CI including 0 indicates that the *UR* values derived from accelerometry did not differ significantly from the ones derived from video analysis. Otherwise, a Δ*UR* above or below 0, respectively, indicated an overestimation or underestimation of the relative use of the MA limb assessed with accelerometry compared to video analysis performed by an experienced clinician. Therefore, this analysis tested for the systematic bias of one method compared to another.

Then, to test the similarity of values obtained with each method (accelerometry vs. video analysis), *intraclass correlation coefficients* (*ICC*) were computed for each epoch length and each AC threshold. An ICC below 0.5 indicates a poor correlation, an *ICC* between 0.5 and 0.75 indicates a moderate correlation, an *ICC* between 0.76 and 0.9 indicates a good correlation, and an *ICC* greater than 0.9 indicates an excellent correlation [37]. Alpha threshold was set at 0.05.

## 3. Results

### 3.1. Sample Description

A total of 29 participants were recruited for the two groups (15 adults and 14 children). Sociodemographic and clinical characteristics are presented in Table 1.

### 3.2. Effect of Epoch Length and Activity Count Threshold on Use Ratios

**Figure 2:**
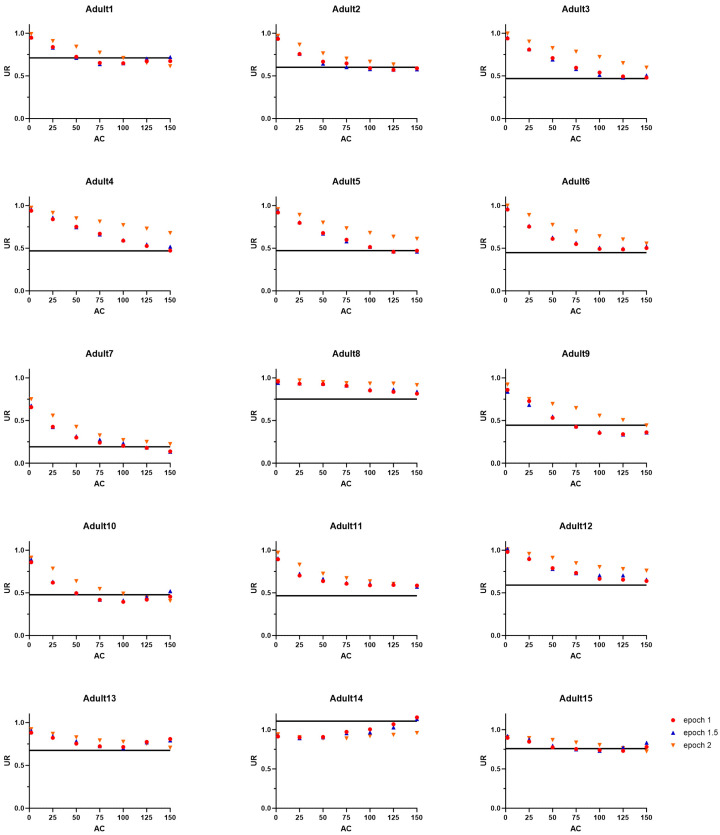
*Use ratio* (*UR*) derived from accelerometry data for each epoch and each activity count (*AC*) *threshold* relative to the *UR* derived from video analysis (represented by the black line) for each participant of the Adult Group.

**Figure 3:**
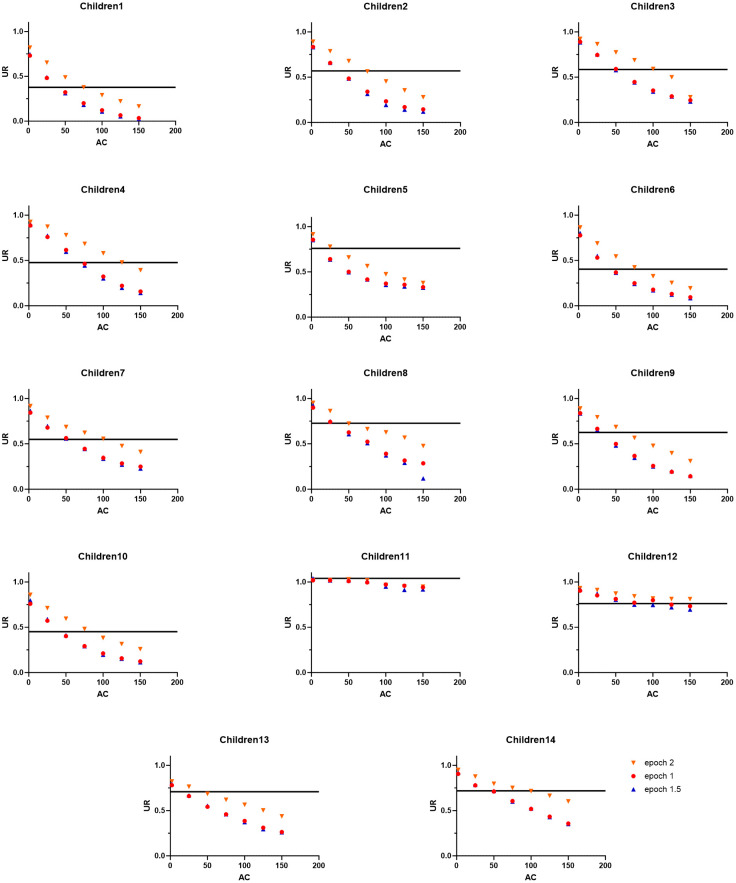
*Use ratio* (*UR*) derived from accelerometry data for each epoch and each activity count (AC) threshold relative to the UR derived from video analysis (represented by the black line) for each participant of the Children Group.

Figure 2 and Figure 3, respectively, show the individual *UR* measured for each combination of parameters in each participant of the Adult Group or Children Group. It can be observed that for the Adult Group (Figure 2), higher *AC thresholds* systematically lead to a better concordance between the *UR* derived from accelerometry data and the one derived from video analysis. This is not the case in the Children Group, in which the combination of parameters that yields the highest concordance with *UR* derived from video analysis is more variable across individuals, falling between *AC thresholds* of 25 to 125 (depending on the epoch length). However, the *AC threshold* of twelve children (except children 11 and 12) out of fourteen falls below 100, showing that the *AC threshold* for children tends to be lower than that for adults.

**Figure 4:**
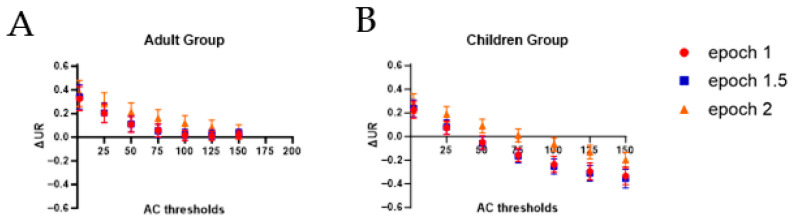
Mean difference between the *use ratio* (*UR*) derived from accelerometry data and the one derived from video analysis (Δ*UR*) for each epoch and for each activity count (*AC*) *threshold* tested. Error bars show the 95% confidence interval. Panel (**A**) presents the data for the Adult Group, and Panel (**B**) for the Children Group.

Figure 4 and Table 2 report group data for each epoch and each *AC threshold*. To account for the fact that everyone has a different *UR*, Δ*UR* were averaged (i.e., the difference between the *UR* derived from accelerometry and the one based on video analysis). Therefore, values close to one indicated concordance between both methods. It can be observed in Figure 4 that for both groups, epoch lengths of 1 and 1.5 provide very similar *UR* estimates, while the epoch length of 2 tends to provide higher *UR* estimates (especially in children). In the Adult Group, the epoch of 1 or 1.5 combined with an *AC threshold* of 100, 125, and 150 presents the smallest difference between the two measurements, with the 95%CI including 0 (see Table 2). For the epoch of 2, the best threshold was an *AC* value of 150. For the Children Group, the best parameters are a combination of an epoch of 1 and 1.5 with an *AC threshold* of 50, or of an epoch of 2 with an *AC threshold* of 75 (see Table 2).

### 3.3. Association between the UR Derived from Accelerometry and the One Derived from Video Rating

Table 3 presents the *ICC* calculated between the *UR* derived from the accelerometry and the one derived from the video rating. In the Adult Group, significant correlations were found for all the combinations of epoch lengths and *AC thresholds*, except for the *AC threshold* of 2. However, good to excellent correlations were observed only for the higher *AC thresholds* (≥75 for epoch lengths of 1 and 1.5, and ≥125 for the epoch length of 2). In the Children Group, most combinations of parameters also yielded significant correlations, but those are moderate at best. The epoch lengths of 1 and 1.5 displayed similar results with the highest association (i.e., moderate) for the *AC threshold* of 50. The epoch length of 2 exhibits the highest association when combined with an *AC threshold of* 75, 100, or 125.

## 4. Discussion

The findings of this study underscore the substantial impact of epoch length and *AC threshold* on the estimates of *UR* in populations with neurological impairments, emphasizing the importance for researchers to carefully select their thresholding method when using accelerometry to assess the relative use of UE. To our knowledge, this is the first study to systematically explore the influence of processing methodology, specifically the combination of different epoch lengths and *AC thresholds*, on UE quantification. While researchers generally acknowledge the need for methodological standardization [15], our results provide valuable insights into identifying the optimal threshold based on the population studied (i.e., adults or children with CP) and the selected epoch length. Both analysis methods (i.e., Δ*UR* and *ICC*) lead to the conclusion that *AC thresholds* of 100 and above are better (i.e., more closely associated to the ratings made by an experienced occupational therapist) when using accelerometry with adults having CP for all the epoch lengths, while the *best AC threshold* for children is lower, ranging from 50 with an epoch length of 1 and 1.5 to 75 with an epoch length of 2. The lower concordance observed between methods in the Children Group can be explained by the larger inter-individual variability.

This recommendation diverges significantly from common practices in the literature, where most studies employ an *AC threshold* of 2 with an epoch length of 1 or 2 [16,38]. The use of an *AC threshold* to quantify UE movements was introduced in [16], which identified a threshold of 2 as the most effective for quantifying UE movement. Their agreement with video recordings was high, with a correlation of 0.93. In contrast, our study found a weak association with our lowest *AC threshold* (i.e., 2), and this disparity may be explained by several factors. Firstly, the method they used for video rating involved 2 s intervals, while we rated movements continuously which is more precise as it encompasses movement transitions. Secondly, they employed different criteria to identify arm movements; participants were required to displace their arm by more than 2.5 cm or perform a pro-supination of more than 30 degrees. This once again reduces the impact of movement transitions, as most disagreements are recorded during small movements. Thirdly, they only provided a few details on the algorithm used to perform *AC* calculation. To address this issue, we developed an open-source algorithm, as detailed in [36], that reproduces a widely used algorithm for *AC* processing (i.e., based on the algorithm of the Actigraph company). Recently, Pohl, Ryser, Veerbeek, Verheyden, Vogt, Luft, and Easthope [24] also questioned the use of a low *AC threshold* based on the use of a machine learning algorithm to identify the best *AC threshold* for each arm in a population of persons having sustained a stroke. An important difference to keep in mind is that in these studies, the selection of the optimal parameter was based on unilateral movement, while in the present study we focused specifically on the *UR*, the metric that is most commonly used to assess the use of the UE. Finally, our results are also similar to the one found in stroke survivors [39,40] as the correlation between the *AC* and the video rating was moderate to good (i.e., *r* = 0.58–0.93) on longer epoch length (i.e., 15 s instead of 1, 1.5, or 2) with an *AC threshold* of 1. Those results were performed during exercise training instead of observing UE utilization during real-life activities, making further comparisons more restricted with our results.

Our study revealed a substantial difference in the optimal thresholding method between adults and children sharing the same neurological condition. This variation could be attributed to several factors, including differences in arm length. Indeed, for a same-arm angular acceleration, linear acceleration measured by an accelerometer is influenced by the lever arm, which is longer for adults than for children. This factor may also account for the higher variability observed in the Children Group compared to the Adult Group. Another factor might be the differences in movement patterns, as adults may develop distinct strategies over time or have less non-functionally relevant movements (e.g., fidgeting). This discrepancy within the same neurological conditions raises questions about the applicability of a uniform threshold for all neurological conditions. Indeed, past studies have often employed thresholds validated for stroke survivors, reflecting the higher prevalence of research in stroke patients compared to individuals with CP. However, distinctions in movement patterns may arise as individuals with CP have lived with their conditions since birth, potentially developing unique compensation patterns compared to stroke patients. Another difference between these two populations is the prevalence of spasticity, which ranges from 20 to 30% for persons having had a stroke and from 72 to 91% for individuals with CP [41,42]. The presence of spasticity in the UE often results in a double flexion arm position (shoulder and elbow flexion). This can influence UE quantification as the arm is maintained close to the body and tends to move when the trunk is in motion.

### Study Limitations

This study has a few limitations. Firstly, the sample size for both groups is small, which could potentially introduce bias to our conclusions. However, the observed heterogeneity in motor deficits within our samples helps mitigate this risk (i.e., participants having mild to moderate UE impairments). Moreover, a sample size of fifteen or lower is often used with this type of methodology, demonstrating that our results are comparable (i.e., see [16,39,40]). Secondly, the tasks used to investigate the effects of epoch length and *AC threshold* on *UR* differ between groups as the data used arise from distinct studies. The tasks performed by the Adult Group were performed standing up/walking and involved fewer fine motor movements compared to those performed by the Children Group. Given that whole-body movements can impact UE quantification [43], differences in methodology may contribute to explain differences between the two groups. Those different protocols were employed to align with the prevailing assessments commonly used in each population. Specifically, the Assisting Hand Assessment is widely recognized as the standard for evaluating bilateral UE utilization in children and the protocol utilized for adults underwent recent validation within this specific population (see [44]). We also validated the data on the *UR* instead of using the amount of *AC* for each arm separately as it has been demonstrated to mitigate the impact of locomotion UE performance [45]. When selecting accelerometry processing parameters, one should consider all environmental factors, and our results should serve as a guide to decide the best parameters. For example, accelerometers are recognized for their proficiency in identifying global movements rather than fine motor movements. Therefore, the compromise between counting the maximum of fine motor movements or reducing the impact of locomotion on UE quantification should be carefully considered by the researcher when selecting their data processing methodology. Concerning epoch length selection, we recommend obtaining raw signals when possible or opting for smaller epochs (e.g., 1 instead of 2), as it allows for modifying the epoch used to match the research question (i.e., if the data recording is at an epoch of 2, it is impossible to use a smaller epoch in offline processing).

## 5. Conclusions

The results of this article highlight the importance of carefully choosing the combination of epoch length, metric, and *AC threshold* when processing accelerometry data for UE quantification, and to consider these factors when comparing studies in the literature. Smaller epoch lengths, such as 1 and 1.5, require smaller *AC thresholds* than the epoch length of 2. Our results also suggest that the height of the participant should be considered in the selection of an appropriate thresholding method. Indeed, our findings suggest that when assessing children, a smaller threshold should be used. Overall, this article challenges the idea of using very small thresholds, such as the one commonly used in the literature, to quantify UE relative use. Our results indicate that they can lead to an overestimation of the relative use of the MA UE.

## Figures and Tables

**Table 1:** Sociodemographic and clinical description of each group of participants.

	Adults (*n* = 15)	Children (*n* = 14)
Age (years; Mean ± SD)	34.5 ± 12.2	10.6 ± 1.9
Sex	6 males/9 females	7 males/7 females
Manual Ability Classification Scale (MACS) level	I = 4	I = 4
	II = 6	II = 5
	III = 5	III = 5
Handedness	10 left-handed	8 left-handed
	5 right-handed *	6 right-handed
Side of lesion	Right = 11 *	Right = 6
	Left = 4	Left = 8
Jebsen Taylor Hand Function Test (Z-score; Mean ± SD)	MA: 23.9 ± 35.0	MA: 50.9 ± 59.5
	LA: 1.4 ± 4.4	LA: 1.61 ± 2.0
*Use ratio* based on video rating (Mean ± SD)	0.58 ± 0.21	0.62 ± 0.18

Legend: MA = more affected; LA = less affected; SD = standard deviation; * one of the participants in the Adult Group was right-handed even if they had a right hemiparesis.

**Table 2:** Mean difference between the *use ratio* (*UR*) derived from accelerometry data and the one derived from video analysis (Δ*UR*) for each group and each *activity count* (*AC*) *threshold*.

Epoch	*AC*	Adults		Children	
		Mean Δ*UR*	95% CI	Mean Δ*UR*	95% CI
1	2	0.33	0.22–0.43	0.23	0.15–0.30
	25	0.20	0.12–0.28	0.08	0.02–0.13
	50	0.11	0.04–0.17	**−0.05**	**−0.11–0.006**
	75	0.06	0.004–0.11	−0.16	−0.21–(−0.10)
	100	**0.02**	**−0.03–0.06**	−0.24	−0.30–(−0.17)
	125	**0.01**	**−0.03–0.05**	−0.29	−0.36–(−0.22)
	150	**0.02**	**−0.01–0.05**	−0.33	−0.41–(−0.26)
1.5	2	0.34	0.24–0.44	0.24	0.16–0.31
	25	0.21	0.13–0.29	0.08	0.02–00.14
	50	0.11	0.06–0.18	**−0.06**	**−0.11–0.0007**
	75	**0.05**	**−0.002–0.11**	−0.17	−0.22–(−0.11)
	100	**0.02**	**−0.03–0.07**	−0.25	−0.32–(−0.19)
	125	**0.02**	**−0.02–0.06**	−0.31	−0.38–(−0.24)
	150	0.03	0.001–0.07	−0.36	−0.44–(−0.28)
2	2	0.37	0.26–0.48	0.28	0.20–0.36
	25	0.28	0.19–0.38	0.19	0.12–0.25
	50	0.21	0.13–0.29	0.09	0.03–0.15
	75	0.16	0.08–0.23	**0.009**	**−0.05–0.096**
	100	0.12	0.05–0.18	−0.07	−0.12–(−0.01)
	125	0.08	0.02–0.15	−0.13	−0.19–(−0.07)
	150	**0.05**	**−0.01–0.11**	−0.20	−0.27–(−0.13)

Legend: *AC* = *activity count*; *UR* = *use ratio;* the combination of factors for which the 95% confidence interval (CI) included 0, meaning that the *UR* derived from accelerometry data did not differ significantly from the one derived from video analysis, appears in bold.

**Table 3:** Intraclass correlation coefficient (ICC) between the use ratio derived from accelerometry and the one derived from video analysis (with *p*-value) for each group, epoch, and AC threshold.

	Adults						Children					
	Epoch 1		Epoch 1.5		Epoch 2		Epoch 1		Epoch 1.5		Epoch 2	
	*ICC*	*p*-Value	*ICC*	*p*-Value	*ICC*	*p*-Value	*ICC*	*p*-Value	*ICC*	*p*-Value	*ICC*	*p*-Value
2	0.31	0.12	0.31	0.12	0.18	0.25	0.58	0.01	0.56	0.01	0.40	0.07
25	0.66	0.003	0.65	0.003	0.48	0.03	0.80	<0.001	0.78	<0.001	0.68	0.003
50	0.79	<0.001	0.79	<0.001	0.64	0.004	0.85	<0.001	0.86	<0.001	0.80	<0.001
75	0.88	<0.001	0.87	<0.001	0.72	<0.001	0.87	<0.001	0.87	<0.001	0.84	<0.001
100	0.93	<0.001	0.91	<0.001	0.79	<0.001	0.84	<0.001	0.85	<0.001	0.86	<0.001
125	0.95	<0.001	0.91	<0.001	0.82	<0.001	0.84	<0.001	0.85	<0.001	0.86	<0.001
150	0.96	<0.001	0.97	<0.001	0.85	<0.001	0.83	<0.001	0.80	<0.001	0.84	<0.001

Legend: Shaded areas indicate that the 95% CI of the Δ*UR* included 0 (see Table 2). Significant *intraclass correlation coefficients* (*ICC*) appear in color. Those below 0.5 (poor correlation) are in red, *ICC* between 0.5 and 0.75 (moderate correlation) are in orange, and *ICC* above 0.76 (good to excellent correlation) are in green. *ICC* that are not statistically significant are in black.

## Data Availability

The data presented in this study are available on request from the corresponding author. The data are not publicly available due to restrictions related to ethical approval.
